# Antibacterial Activity of Nanomaterials

**DOI:** 10.3390/nano8060359

**Published:** 2018-05-24

**Authors:** Ana María Díez-Pascual

**Affiliations:** Department of Analytical Chemistry, Physical Chemistry and Chemical Engineering, Faculty of Biology, Environmental Sciences and Chemistry, Institute of Chemistry Research “Andrés M. del Río” (IQAR), University of Alcalá, Ctra. Madrid-Barcelona, Km. 33.6, 28871 Alcalá de Henares, Madrid, Spain; am.diez@uah.es; Tel.: +34-918-856-430

Bacterial adhesion and proliferation is a serious and increasing concern in everyday life, and is responsible for significant damage in several industries, including textile, water treatment, marine transport, medicine and food packaging. Notwithstanding the enormous efforts by academic researchers and industry, a general solution for restricting bacterial colonization has not been found yet. Therefore, new strategies for controlling bacterial activity are urgently needed, and nanomaterials constitute a very promising approach. This Special Issue, with a collection of 21 original contributions and one commentary, provides selected examples of the most recent advances in the synthesis, characterization and applications of nanomaterials with antibacterial activity.

Silver nanoparticles (AgNPs) are well-known antibacterial agents versus a broad spectrum of Gram-positive and Gram-negative bacteria, including antibiotic-resistant strains. They have gained a lot of interest owing to their chemical stability, catalytic activity, wound-healing capacity, high conductivity and surface plasma resonance. It has been demonstrated that they possess higher antibacterial activity compared with their bulk counterparts owing to their higher surface-to-volume ratio, providing better contact with microorganisms [1]. For instance, Al-Dhabi et al. [2] synthesized AgNPs from active marine *Streptomyces* sp. Al-Dhabi-87 collected from Dammam and Al-Kohbar regions of Saudi Arabia. The NPs showed noticeable antimicrobial activity against wound-infection pathogens such as *Bacillus subtilis*, *Enterococcus faecalis*, *Staphylococcus epidermidis*, multidrug-resistant *Staphylococcus aureus* and *Escherichia Coli* strains.

However, the low stability of AgNPs hinders some medical or hygienic applications, and hence it is important to investigate the shelf life of the material under different storage conditions. Korshed et al. [3] investigated the antibacterial effects of laser-grown AgNPs, stored under different conditions (daylight, dark and cold), against *E. Coli* bacteria. Results showed that the antibacterial activity of the laser-generated AgNPs lasted 266 to 405 days, over 100 days longer than the chemically produced ones. Another concern is the potential toxicity of AgNPs in the environment and in human beings, since it has been reported [4] that they induce damage in mitochondrial cells of a number of organisms, including mammals. A simple solution is the use of zeolites, in which Ag^+^ can be incorporated by ionic exchange. The idea is not only to introduce Ag^+^ inside a solid matrix, but also to form bonds that hinder the release of Ag^+^ from the material. In this regard, Jędrzejczyk et al. [5] investigated the antimicrobial activity of Ag cations trapped in a faujasite-type zeolite and added to paper pulp to obtain sheets. The paper with the modified faujasite additive showed higher antibacterial activity towards *E. Coli*, *Serratia marcescens*, *B. subtilis* and *Bacillus megaterium*, as well as better antifungal action against *Chaetomium globosum*, *Cladosporioides* and *Aspergillus niger* than AgNP-filled paper. Another method for immobilizing AgNPs is their incorporation in a silica matrix to form Ag–SiO_2_ nanocomposites [6]. A gauze impregnated with a Ag–SiO_2_ sample showed higher antibacterial effects against *S. aureus* and *E. Coli* than commercial Ag-containing dressings, indicating their suitability for the management and infection control of superficial wounds.

An alternative approach to reduce the possible toxicity of AgNPs and improve their efficacy and stability in biomedical applications is the use of nontoxic and noninflammatory capping agents like collagen, peptides and biopolymers. Thus, Tanvir et al. [7] compared the antibacterial properties of AgNPs with different morphologies: spheres and prisms, stabilized by polyvinylpyrrolidone and coated with poly-l-arginine. NPs with prismatic morphology exhibited stronger antimicrobial effects against *E. Coli*, *Pseudomonas aeruginosa* and *Salmonella enterica*, and also had noteworthy cytotoxic effects towards an in-vitro HeLa cancer cell line.

The combination of AgNPs with other nanomaterials with antibacterial activity such as graphene oxide (GO) leads to enhanced antibiotic properties due to synergistic effects. The 2D layered structure of GO can wrap the bacterial cell membrane and cause oxidative stress at the basal plane, thus damaging the cellular membrane. Tuong Vi et al. [8] investigated the antibacterial activity of Ag–GO nanocomposites covalently grafted via thiol groups, without the need for reducing agents or stabilizers. The sample with 43% Ag demonstrated the highest antibacterial efficiency, about 100% against *S. aureus* and *P. aeruginosa*. The proposed antimicrobial mechanism is that the GO wraps around bacteria while the Ag kills the bacteria with its toxicity. The nanodiamond (ND) is another type of carbon nanomaterial that hinders the growth of microorganisms. Jira et al. [9] compared the effect of ND and GO under both annealed (oxidized) and reduced (hydrogenated) forms on two types of cultivation media: Luria-Bertani and Mueller-Hinton broths. A noteworthy long-term inhibition of *E. Coli* growth was only found by hydrogenated ND in the Mueller-Hinton medium. 

AuNPs also exhibit significant antimicrobial activity. Nonetheless, the actual methods for NP production are frequently expensive and use chemicals that are potentially harmful to the environment. In this context, Aljabali et al. [10] reported the synthesis of AuNPs using plant extracts, in particular the Ennab leaves, which have antifungal, antibacterial, antiulcer and anti-inflammatory activities. This alternative, simple, fast, low-cost and environmentally friendly method produces NPs with different geometries: spherical, triangular, hexagonal platelets and truncated, and opens up the possibility for using AuNPs in drug delivery, oral or intranasal, without interfering with the human microbiota. The biogenic synthesis of AuNPs is another green and nontoxic approach to produce biocompatible NPs for biomedical applications. In this regard, Elbagory et al. [11] prepared biogenic AuNPs from the *Galenia Africana* and *Hypoxis hemerocallidea* South African plant extracts and examined their effect against bacterial strains that provoke wound infections. The NPs did not show any toxicity towards cancerous human fibroblast cells (KMST-6), hence are promising candidates for wound-dressing applications. 

Recently it has been found that TiO_2_ NPs have a broad spectrum of activity against microorganisms, including Gram-negative and -positive bacteria and fungi, which is highly interesting for multiple-drug-resistant strains [12]. More importantly, TiO_2_-based nanocomposites are environmentally friendly and exert a noncontact biocidal action. Therefore, no release of potentially toxic nanoparticles to the medium is necessary to attain disinfection capabilities. Milosevic et al. [13] developed a simple, cost-effective and scalable wet milling method to prepare fluorinated and N-doped TiO_2_ nanopowders with improved photocatalytic properties under visible light. Further, the combination of N and F led to better activity against *E. Coli* due to synergistic effects. These novel nanomaterials are adequate for biomedical applications, such as hospital tools, but also food preservation or wastewater treatment. 

Moreover, Fe_3_O_4_ NPs can exhibit antimicrobial activity and act synergistically with other antimicrobial substances for the controlled release of antimicrobial agents. In this regard, Limban et al. [14] synthesized hybrid nanosystems based on 2-((4-chlorophenoxy)methyl)-*N*-(substituted phenylcarbamo-thioyl)benzamides and Fe_3_O_4_@C_18_ core@shell nanoparticles. The C_18_ acts as a spacer and facilitates the interaction between the magnetite nanoparticles and the thiourea derivatives. The hybrids showed good biocompatibility and high efficiency in preventing the development of *C.* *albicans* biofilms, hence are promising nanocarriers of antifungal substances for therapeutic and prophylactic use, effective on both planktonic and biofilm-embedded cells.

Biopolymers are also excellent candidates for the preparation of antimicrobial nanomaterials. In particular, positively charged chitosan can interact with negatively charged cell membranes, resulting in alterations in the cell wall permeability and the leakage of intracellular compounds. Nonetheless, parameters like molecular weight, deacetylation degree and positive-charge content strongly influence the bactericidal action of this polysaccharide [15]. Some studies have modified chitosan with sulfonate groups or quaternary ammonium groups, and integrated antibacterial herbs or enzymes into chitosan beads or nanoparticles to enhance their antimicrobial activities. Tamara et al. [16] developed chitosan NPs comprising protamine, a natural cationic antimicrobial peptide composed of arginine residues. The hybridization of chitosan with protamine boosted the antibacterial activity of chitosan nanoparticles towards pathogenic *E. Coli*, but the inhibitory effect against probiotic *B. cereus* was considerably reduced. Sanmugam et al. [17] prepared chitosan–ZnO and chitosan–ZnO–GO hybrid nanocomposites using a one-pot chemical strategy, and their dye adsorption characteristics, and thermal, mechanical and antibacterial properties, were investigated. The hybrids displayed better thermal stability, mechanical strength, flexibility, and stronger antibacterial activity against *E. Coli* and *S. aureus*. They also showed good dye adsorption behavior for methylene blue and chromium complex. Therefore, they are suitable candidates for bacterial growth inhibition and absorption of toxic dyes in water treatment, food packaging, adhesives, tissue engineering, and medical and pharmaceutical applications. 

Cationic NPs of polystyrene sulfate covered by a bilayer of dioctadecyldimethylammonium bromide incorporating an antimicrobial peptide, gramicidin, were prepared by Xavier and Carmona-Ribeiro [18], leading to strong bactericidal activity against both *E. Coli* and *S. aureus* at very small concentrations of the antimicrobials. These results corroborate the advantages of highly organized, cationic hybrid nanoparticles that combine polymers and peptides. Furthermore, biopolymer NPs of polylactide-co-glycolide and polylactide-co-glycolide-co-polyethylenglycol blends loaded with gentamicin, an aminoglycoside antibiotic used to treat bacterial infections, have been developed by Dorati et al. [19]. A screening design was applied to optimize the drug load, NP size and size distribution, and stability and resuspendability after freeze-drying. 

Polymeric composites comprising NPs such as ZnO have been widely investigated as food-packaging materials. In this context, the antibacterial action of poly (lactic acid)-based electrospun mats incorporating ZnO-NPs and mesoporous silica doped with ZnO was assessed by Rokbani et al. [20]. A concentration-dependent effect of these nanomaterials on the viability of *E. Coli* was demonstrated. Moreover, the combination of the ultrasound stimulations and autoclave sterilization considerably enhanced the antimicrobial activity of the electrospun mats. Another study [21] demonstrated that cellulose-based packaging materials comprising polyethylene films reinforced with ZnO nanoparticles were very active against mesophilic and psychotropic bacterial cells. 

Gelatin is a biodegradable polymer with immense industrial applications due to its gelling properties, ability to form and stabilize emulsions, its adhesive properties, and dissolution behavior. Figueroa-Lopez et al. [22] mixed gelatin with polycaprolactone as a barrier coating and black pepper oleoresin as a natural extract using the electrospinning coating technique in order to improve the antimicrobial properties and the water-vapour barrier performance. The hybrid materials showed lower wettability, improved water barrier and flexibility as well as stronger antimicrobial behavior against *S. aureus*, and show great potential for use in active food-packaging applications.

In addition to polymeric materials, lipid-based nanostructures have also been developed as promising nanosized drug carriers, for the delivery of antibacterial, antifungal and antiviral drugs. For such purposes, Pignatello et al. [23] synthesized solid lipid nanoparticles loaded with ciprofloxacin, a bactericidal antibiotic highly effective against Gram-positive and Gram-negative bacteria, frequently used in urinary tract infections. The synthesis was carried out via two different techniques: quasi-emulsion solvent diffusion and solvent injection, using the cationic lipid didecyldimethylammonium bromide. Homogeneous lipid nanoparticles with sizes in the range of 250–350 nm were produced by both methods, which were stable up to nine months both at 4 °C and 25 °C, and their encapsulation efficiency was higher than 85%. 

Natural essential oils are becoming popular antimicrobials due to their phenolic compounds, and have been proposed as efficient, environmentally friendly, economic and nontoxic acaricides to humans in the indoor environment [24]. However, most essential oils are volatile or easily oxidized, which limits their practical use. A convenient solution to the problem is their microencapsulation. In this context, Kim and Kim [25] developed acaricidal electrospun nylon 66 nanofibers grafted with clove oil-loaded microcapsules. The increase in the microcapsule content from 5 to 15 wt % significantly increased the mortality against *Dermatophagoides farinae*. Therefore, eco-friendly clove oil can be used as efficient replacement for synthetic acaricides in controlling the population of common indoor house dust-mite species.

Another interesting application of antimicrobial nanoparticles, including metals and oxides, can be found in building materials (especially cement-based composites). However, apart from the known toxicity of nanomaterials, in the case of cement-based composites there are limitations related to the mixing and dispersion of nanomaterials. In this regard, Sikora et al. [26] tested the antibacterial activity of different nanooxides (Al_2_O_3_, CuO, Fe_3_O_4_ and ZnO) against a number of microorganisms, and found that metal oxide nanoparticles could not be efficient for hindering microbial growth when not properly dispersed, which will likely be the case in cement mortars and concretes. Thus, new methods for improving the dispersion of nanomaterials are sought.

Finally, it is worthy to highlight that despite much interest in using nanomaterials for a range of antimicrobial applications in agriculture and medicine, most of this work has been carried out by engineers and chemists who take into account how biological systems react to novel materials on either a physiological or an evolutionary timescale. In this regard, Graves Jr. et al. [27] commented on how combined approaches that employ both the variety of elements (Ag, Cu, Fe, Au, fullerenes, etc.) and biologics (bacteriophage and antibiotic compounds), as well as shapes (nanoplates, nanorods, nanodarts, etc.), provide the best chance to design sustainable antimicrobial nanomaterials.
